# Boundary Layer Flow Past a Stretching Surface in a Porous Medium Saturated by a Nanofluid: Brinkman-Forchheimer Model

**DOI:** 10.1371/journal.pone.0047031

**Published:** 2012-10-15

**Authors:** Waqar A. Khan, Ioan M. Pop

**Affiliations:** 1 Department of Engineering Sciences, PN Engineering College, National University of Sciences and Technology, Karachi, Pakistan; 2 Faculty of Mathematics, University of Cluj, Cluj, Romania; US Naval Reseach Laboratory, United States of America

## Abstract

In this study, the steady forced convection flow and heat transfer due to an impermeable stretching surface in a porous medium saturated with a nanofluid are investigated numerically. The Brinkman-Forchheimer model is used for the momentum equations (porous medium), whereas, Bongiorno’s model is used for the nanofluid. Uniform temperature and nanofluid volume fraction are assumed at the surface. The boundary layer equations are transformed to ordinary differential equations in terms of the governing parameters including Prandtl and Lewis numbers, viscosity ratio, porous medium, Brownian motion and thermophoresis parameters. Numerical results for the velocity, temperature and concentration profiles, as well as for the reduced Nusselt and Sherwood numbers are obtained and presented graphically.

## Introduction

Flow in porous media has been the subject of numerous investigations during the past several decades. The interest in this subject has been stimulated, to a large extent, by the fact that thermally driven flows in porous media have several applications in chemical and mechanical engineering, e.g. food processing and storage, geophysical systems, electro- chemistry, fibrous insulation, metallurgy, the design of pebble bed nuclear reactors, underground disposal of nuclear or non-nuclear waste, microelectronics cooling, etc. Detailed literature review can be found in the books by Pop and Ingham [1], Ingham and Pop [2], Nield and Bejan [3], Vafai [4], [5] and Vadasz [6]. One of the fundamental problems in porous media is the flow and heat transfer driven by a linearly stretching surface through a porous medium. It seems that the first study of the steady flows of a viscous incompressible fluid (non-porous media) driven by a linearly stretching surface through a quiescent fluid has been reported by Crane [7]. Further, Elbashbeshy and Bazid [8] studied flow and heat transfer in a porous medium over a stretching surface with internal heat generation and suction/blowing when the surface is held at a constant temperature. Cortell [9] has presented an analytical solution of the problem considered by Elbashbeshy and Bazid [8] considering the following two cases: (i) constant surface temperature (CST) and (ii) prescribed surface temperature (PST). Extension of these problems were further considered by Pantokratoras [10], Tamayol et al. [11], and Fang and Zhang [12]. Further, we notice that Kaviani [13] has investigated the effect of the solid matrix on the forced convection boundary layer flow and heat transfer from a semi-infinite flat plate embedded in a porous medium. Kaviani [13] transformed the governing equations and solved numerically.

Solid particles can be added in the base fluids of lower thermal conductivity to improve heat transfer. Such fluids were introduced by Choi [14] and are known as nanofluids. These fluids have higher thermal conductivity and thus give higher thermal performance. It was shown that metallic nanoparticles with high thermal conductivity increase the effective thermal conductivity of these fluids remarkably. Eastman *et al.* [15] showed that an increase in the thermal conductivity depends on the shape, size and thermal properties of the nanoparticles. Several studies have also been reported in the literature, which claim that the addition of nanoparticles in the base fluid may cause a considerable decrease in the heat transfer (Putra *et al.* [16], and Wen and Ding [17]). It is important to note that, in the numerical studies, the increase in the heat transfer depends on the existing models used to predict the properties of the nanofluids (Ho *et al.* [18] and Abu-Nada [19]). Buongiorno [20] found that the nanoparticle absolute velocity can be written as the sum of the base fluid velocity and the slip velocity. Several numerical and experimental studies on the heat transfer using nanofluids are available in the open literature, e.g. Khanafer *et al.* [21], Maiga *et al.* [22], Tiwari and Das [23], Oztop and Abu-Nada [24], Muthtamilselvan *et al.* [25], Ghasemi and Aminossadati [26], Popa et al. [27], etc. The book by Das *et al.* [28] and the review papers by Daungthongsuk and Wongwises [29], Wang and Mujumdar [30], [31], and Kakaç and Pramunjaroenkij [32] present excellent information on nanofluids.

Nield and Kuznetsov [33] revisited the Cheng and Minkowycz’s problem [34] for natural convective boundary layer flow over a vertical flat plate embedded in a porous medium filled with nanofluid. They employed Buongiorno [20] model and considered the combined effects of both heat and mass transfer. In an another paper, Kuznetsov and Nield [35] used the same Buongiorno’s [20] model and obtained numerical solution for the natural convective heat transfer of a nanofluid past a vertical flat plate. Later on, Khan and Pop [36], and Bachock et al. [37] used Buongiorno’s [20] nanofluid model and investigated the boundary-layer flow of a nanofluid past a stretching surface, while Ahmad and Pop [38] investigated the mixed convection boundary layer flow over a vertical flat plate embedded in a porous medium saturated with a nanofluid. They employed the model proposed by Tiwari and Das [23]. Therefore, the present investigation deals with the steady forced convection flow and heat transfer due to a stretching flat surface in a porous medium saturated with a nanofluid by considering the Brinkman-Forchheimer model (see Nield and Bejan, [3]) for the momentum equation and Bongiorno’s [20] model for the energy and nanofluid volume fraction equations. The paper uses, in fact, the idea of the paper by Kuznetsov and Nield [39] to the case of a stretching surface in a nanofluid. The boundary layer equations are transformed to ordinary differential equations in terms of the governing parameters including Prandtl and Lewis numbers, viscosity ratio, porous medium, Brownian motion and thermophoresis parameters. Numerical results for velocity, temperature and concentration profiles, as well as for the reduced Nusselt and Sherwood numbers are obtained and presented graphically for different values of the governing parameters. It is found that these parameters have substantial effects on the flow and heat transfer characteristics.

## Basic Equations

**Figure 1:**
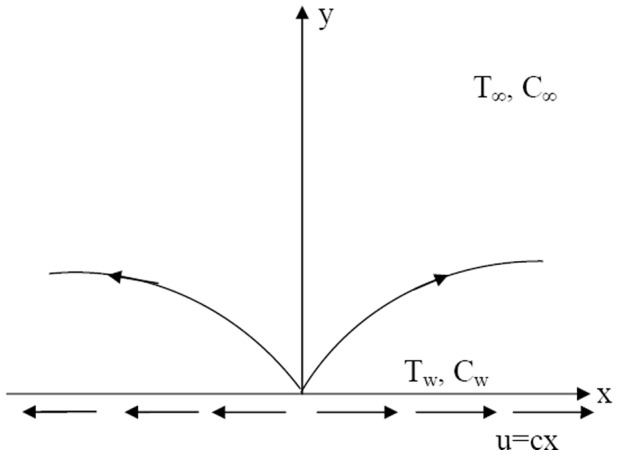
Flow of nanofluid over a stretching sheet.

Consider the steady boundary layer flow past a stretching surface in a porous medium filled with a nanofluid as shown in Fig. 1. It is assumed that the uniform temperature of the surface is 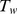 and that of the nanofluid volume fraction is 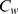, while the uniform temperature and nanofluid volume fraction in the ambient fluid (inviscid flow) are 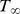 and 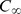, respectively. It is also assumed that the plate is stretched with a linearly velocity 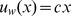, where 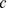 is a positive constant. Further, it is assumed that the second-order inertial term in the Navier-Stokes equations is neglected (see Vafai and Tien [40]; Hong et al. [41], Laurial and Prasad [42], Nakayama [43], Nield and Bejan, pp. 16, [3]). The flow is assumed to be slow so that an advective term and a Forchheimer quadratic drag term do not appear in the momentum equations. Under these assumptions, the following five field equations embody the conservation of total mass, momentum (Brinkman-Forchheimer equations), and energy and nanofluid volume fraction equations for the nanofluid are considered as,

**Figure 2:**
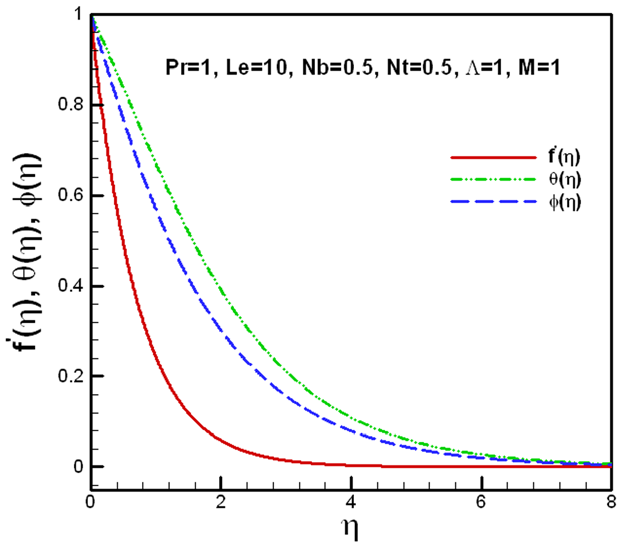
Dimensionless velocity, temperature and concentration profiles for a nanofluid.

**Figure 3:**
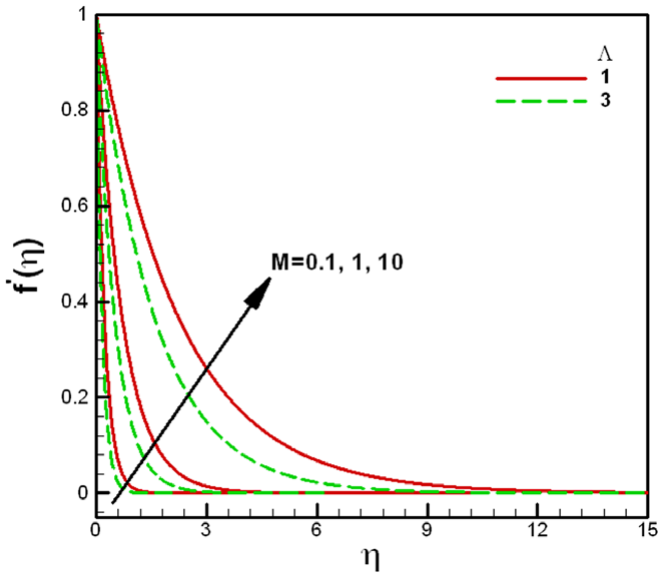
Dimensionless velocity profiles for different values of the viscosity ratio 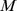 and Porous medium parameters 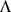.



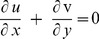
(1)

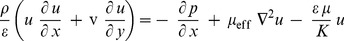
(2)

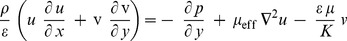
(3)

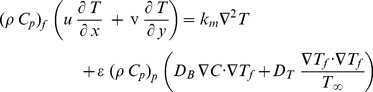
(4)

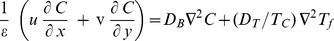
(5)where 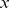 and 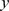 are Cartesian coordinates along the stretching wall and normal to it, respectively, 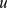 and 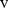 are the velocity components along the 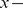 and 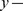 axes, 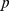 is the pressure, 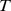 is the temperature in the fluid phase, 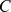 is the nanoparticle volume fraction, 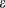 is the porosity, 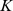 is the permeability of the porous medium, 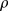 and 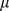 are the density and dynamic viscosity of the fluid, respectively. Further, 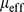 is the effective viscosity, 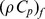 is the heat capacity of the fluid, 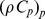 is the effective heat capacity of the nanoparticle material and 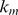 is the effective thermal conductivities of the porous medium. A detailed discussion on 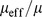 can be found in Nield and Bejan [3]. The coefficients that appear in Eqs. (4) and (5) are the Brownian diffusion coefficient 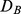 and the thermophoretic diffusion coefficient 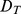. Details of the derivation of Eqs. (4)–(7) are given in the papers by Buongiorno [20], Nield and Kuznetsov [33], Kuznetsov and Nield [39]. The boundary conditions of Eqs. (1)–(5) are



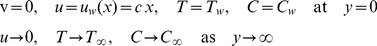
(6)We look for a similarity solution of Eqs. (1)–(4) of the following form.
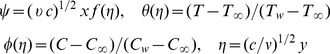
(7)where 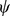 is the stream function which can be defined as 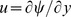 and 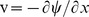. Using (7), Eqs. (2)–(5) can be written as



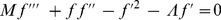
(8)

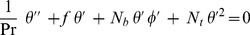
(9)

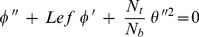
(10)subject to the boundary conditions (6), which become



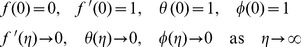
(11)Here primes denote differentiation with respect 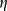 and the six parameters are defined as
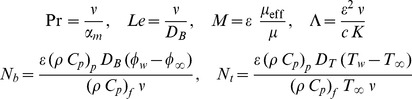
(12)where 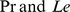 are the Prandtl and Lewis numbers, 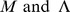 are the viscosity ratio and porous medium parameters, and 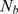 and 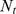 are the nanofluid parameters. It is important to note that this boundary value problem reduces to the classical problem of flow and heat and mass transfer due to a stretching surface in a viscous (regular) fluid when 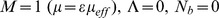 and 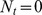 in Eqs. (8)–(10). (The boundary value problem for 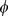 then becomes ill-posed and is of no physical significance). It is worth pointing out that because this is a forced convection problem, Eq. (8) is decupled by Eqs. (9) and (10).

**Figure 4:**
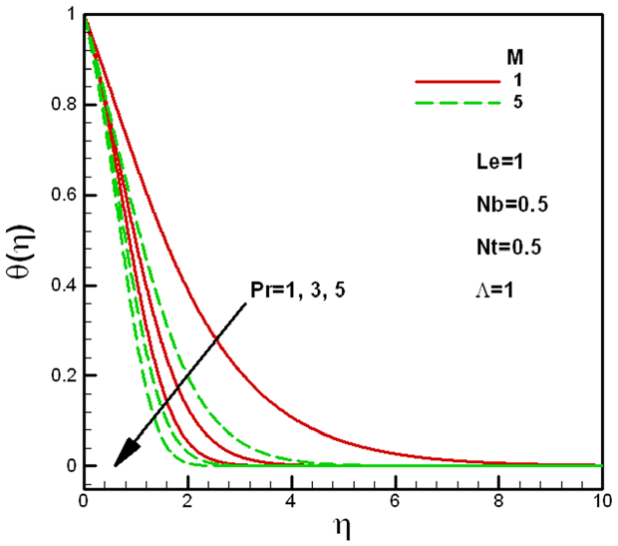
Effects of Prandtl number and the viscosity ratio on dimensionless temperature.

**Figure 5:**
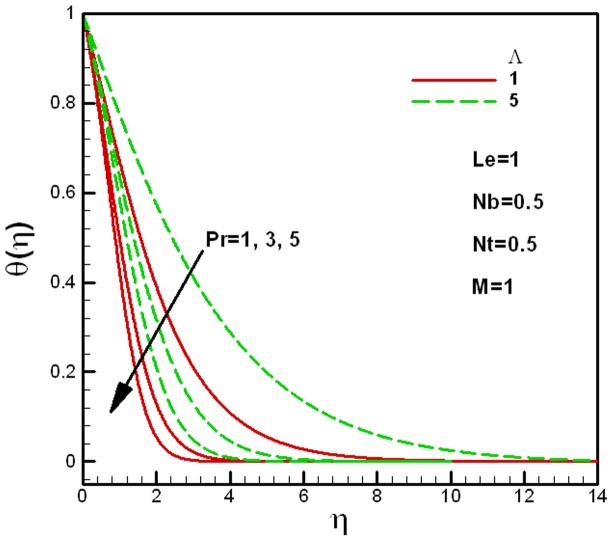
Effects of Prandtl numbers on dimensionless temperature for different values of the porous medium parameter 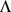.

The physical quantities of most interest are the local Nusselt number 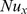 and the local Sherwood number 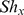, which are defined as
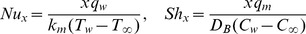
(13)where 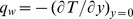 and 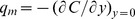 are the heat and mass fluxes from the surface of the sheet. After some algebra, we obtain
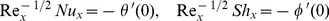
(14)where 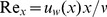 is the local Reynolds number. Kuznetsov and Nield [35] referred 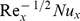 and 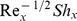 as the reduced Nusselt number 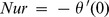 and reduced Sherwood number 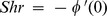, respectively.

We notice that for 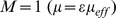 and 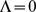 Eqs. (8)–(10) with the boundary conditions (11) reduce to those derived by Khan and Pop [36] for a stretching sheet in a nanofluid. Further, for 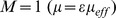, Eq. (8) reduces to
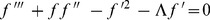
(14)along with the boundary conditions



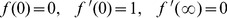
(15)The analytical solution of this problem is given (see Cortell [9]) in the following form
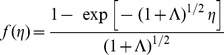
(16)


**Figure 6:**
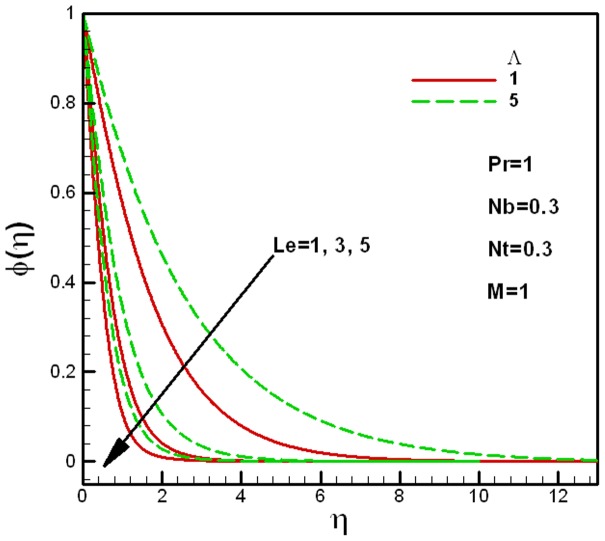
Effects of Lewis numbers on dimensionless concentration for different values of the porous medium parameter 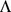.

**Figure 7:**
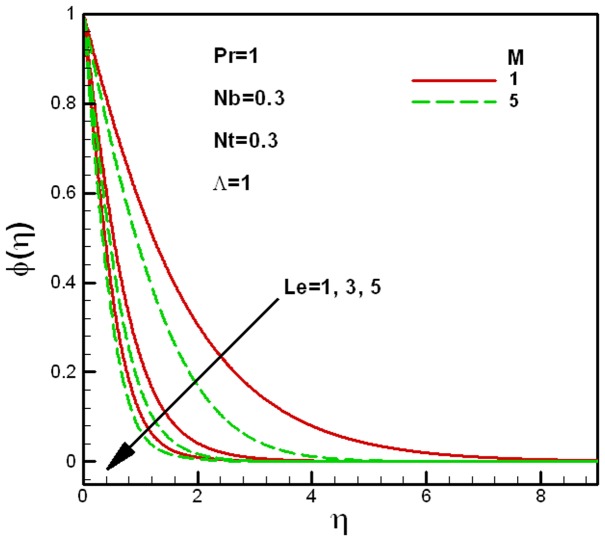
Effects of Lewis numbers on dimensionless concentration for different values of the viscosity ratio parameter 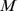.

## Results and Discussion

Equations (8)–(10) with the boundary conditions (11) were solved numerically for different values of the governing parameters where 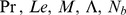 and 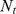 using an implicit finite-difference method as in Khan and Pop [36]. The boundary conditions in Eq. (11) at 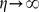 are replaced by a sufficiently large value 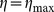. In this study, we get 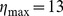 for all values of the governing parameters. The step size of 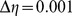 is taken in all cases.

**Figure 8:**
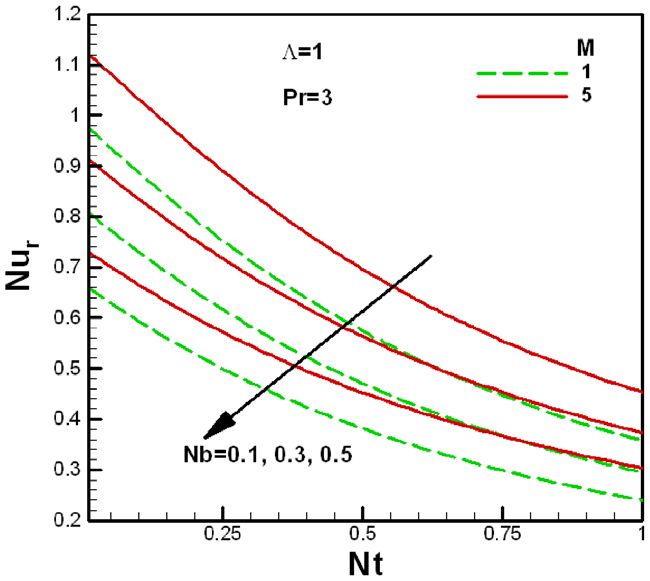
Variation of the reduced Nusselt number with Brownian motion and viscosity ratio parameters.

**Figure 9:**
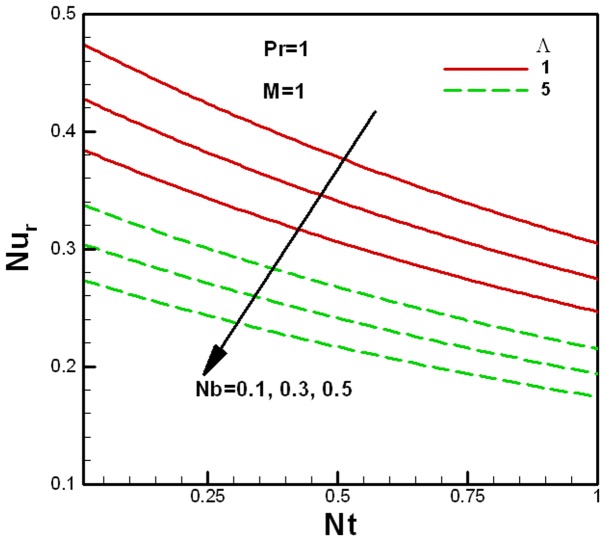
Variation of the reduced Nusselt number with Brownian motion and porous medium parameters.

The velocity, temperature and concentration profiles of a nanofluid for specific conditions are shown in Fig. 2. It is clear from the figure that the velocity converges quickly, whereas the temperature and concentration profiles behave in the same manner and converge together. The effects of the viscosity ratio 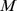 and porous medium parameter 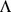 on the velocity profiles are shown in Fig. 3. It is clear from figure that the dimensionless velocity increases and the rate of convergence decreases with an increase in the effective viscosity of the nanofluid. The dimensionless velocity also increases with the decrease in the porous medium parameter 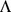. As expected, the dimensionless velocity boundary layer thickness increases with an increase in the porous medium parameter 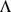 and decrease in the viscosity ratio *M*.

**Figure 10:**
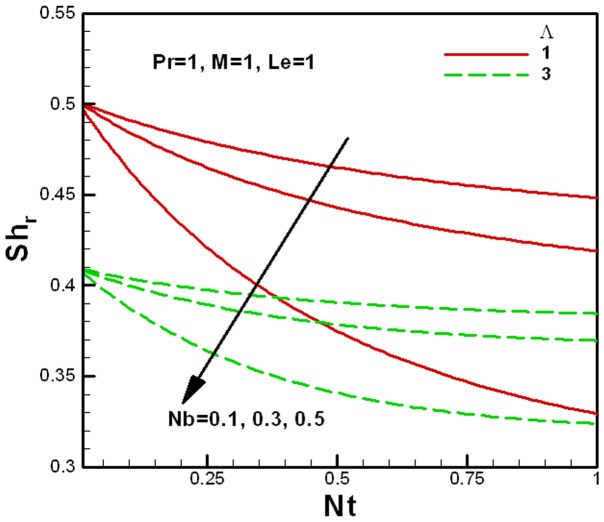
Variation of the reduced Sherwood number with Brownian motion and porous medium parameters.

**Figure 11:**
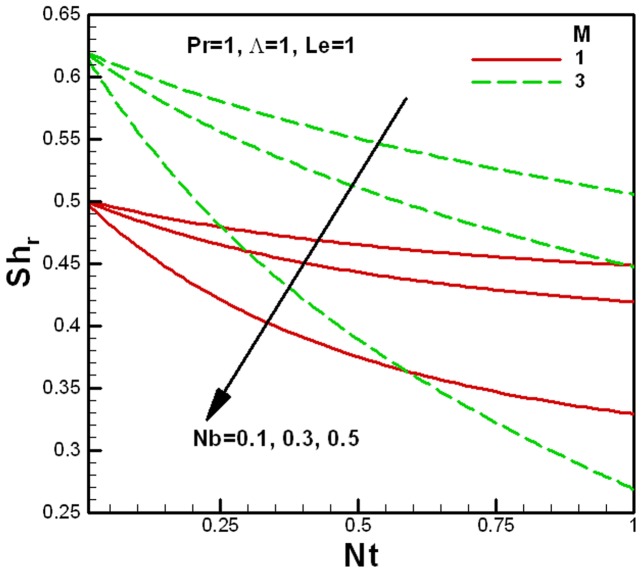
Variation of the reduced Sherwood number with Brownian motion and the viscosity ratio parameters.

Figures 4 and 5 show the effects of Prandtl numbers on temperature profiles for different values of the viscosity ratio 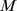
and the porous medium parameter 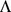 respectively. The other governing parameters like 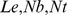 are kept constant. As expected, the thermal boundary layer thickness increases with the decrease in Prandtl number in both figures. Figure 4 shows that the dimensionless temperature increases with the decrease in the viscosity ratio 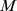, whereas, Fig. 5 shows that the dimensionless temperature increases with the increase in the porous medium parameter 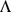. This is actually due to the decrease in the effective viscosity of the nanofluid and the permeability of the porous medium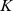.

The effects of the Lewis number 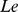 on concentration profiles for different values of the porous medium parameter 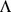 and the viscosity ratio 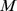 are shown in Figs. 6 and 7 respectively. It can be seen that the concentration boundary layer thickness increases with an increase in the Lewis number. This is due to the fact that the decrease in the Brownian diffusion coefficient 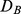
causes an increase in the concentration. Figure 6 shows that the dimensionless concentration increases with an increase in the porous medium parameter 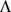. The dimensionless concentration is maximum at the stretching surface and converges quickly for larger values of the Lewis number and porous medium parameter. The dimensionless concentration also converges quickly for larger values of viscosity ratio parameter 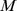, as shown in Fig. 7.

The variation of the reduced Nusselt number 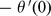 with nanofluid parameters for several values of the viscosity ratio parameter 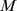 and the porous medium parameter 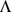 is shown in Figs. 8 and 9 respectively. It can be seen that the reduced Nusselt number decreases with an increase in the Brownian motion and thermophoresis parameters. For the fixed value of the porous medium parameter 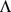, the reduced Nusselt number increases with an increase the viscosity ratio parameter 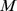, as shown in Fig. 8, whereas the reduced Nusselt number decreases with an increase in the porous medium parameter 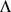. This is shown in Fig. 9 for the fixed value of the viscosity ratio parameter 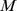.

Figures 10 and 11 show the variation of the reduced Sherwood number 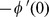 with nanofluid parameters for several values of the porous medium and the viscosity ratios parameters respectively. Like the reduced Nusselt numbers, the reduced Sherwood numbers also decrease with an increase in the Brownian motion and thermophoresis parameters. For smaller values of the Brownian motion and thermophoresis parameters, the change in the reduced Sherwood numbers is smaller but it increase quickly with an increase in 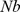 and 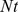, as shown in Fig. 10 for the fixed value of the Lewis number. It also shows that the reduced Sherwood number decreases with an increase in the porous medium parameter 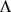. Finally, Fig. 11 shows the effect of the viscosity ratio parameter 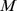
on the reduced Sherwood number for the fixed value of Lewis number. The reduced Sherwood number increases with the viscosity ratio parameter 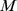.

### Conclusions

In this study, the steady forced flow and heat transfer due to an impermeable stretching surface in a porous medium saturated with a nanofluid are investigated numerically by using an implicit finite difference method. The effects of the viscosity ratio 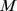 and porous medium parameter 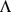 on the dimensionless velocity, temperature and concentration profiles as well as on the reduced Nusselt and Sherwood numbers are presented graphically.
